# “Emotional Proximity” and “Spatial Proximity”: Higher Relationship Quality and Nearer Distance Both Strengthen Scratch Contagion in Tibetan Macaques

**DOI:** 10.3390/ani12162151

**Published:** 2022-08-22

**Authors:** Yu-Heng Zhang, Xi Wang, Meng-Meng Chen, Yi-Mei Tai, Jin-Hua Li

**Affiliations:** 1 School of Resources and Environmental Engineering, Anhui University, Hefei 230601, China; 2 International Collaborative Research Center for Huangshan Biodiversity and Tibetan Macaque Behavioral Ecology, Hefei 230601, China; 3 School of Life Sciences, Hefei Normal University, Hefei 230601, China

**Keywords:** behavioral contagion, empathy, contagious scratching, social centrality, social relationship quality

## Abstract

**Simple Summary:**

In social situations, people may unconsciously repeat their partners’ small moves. Sometimes we can not help opening our mouths when we watch others yawn; other times, we subconsciously cross our legs like others. This phenomenon also exists in animals. We believe that this retained habit is beneficial to social animals. By observing a group of macaques, we found that close partners are more prone to have behavioral synchronization, which is conducive to the maintenance of the group.

**Abstract:**

Behavioral contagion has been defined as a phenomenon in which an unlearned behavior automatically triggers a similar behavior in others. Previous studies showed that a behavioral contagion might have the function of strengthening social relationships, promoting group coordination and maintaining social cohesion. However, so far, there are few studies investigating the correlation between contagious scratching and social bonding. Tibetan macaques (*Macaca thibetana*) live in multi-male and multi-female cohesive matrilineal groups, and scratching is usually observed in their affiliated interactions. We investigated the process of scratch contagion in one group of free-ranging Tibetan macaques and explored whether behavioral contagion could consolidate social relationships and maintain social stability. Results showed that the scratching was contagious and correlated with relationship quality and spatial distance. In dyads with a higher Dyadic Composite Sociality Index (DSI), the contagion was strong. In addition, contagions occurred more frequently and faster among individuals nearer to each other. In terms of social groups, members with higher social centrality participated in more behavioral contagion, whether as expressers or observers. Our findings provide new perspectives for studying behavioral contagions in humans and animals.

## 1. Introduction

In group-living animals, synchronization of behaviors is often observed, which looks like the social glue that holds groups together [1,2]. This phenomenon is also very common in human social interaction and is called “the chameleon effect” [3]. Such unconscious behavioral mimicry has evolved to serve a social function; that is, mimicry increases subordination, which helps to strengthen social relationships between experimenters and subjects [4]. In some primates, this behavior matching effect is also functionally related to social relations. Perhaps, in the constantly running social network, just like matching parts, group members instinctively synchronize with each other to maintain the advantages of the social group and ensure the development of the collective. Nonetheless, the mechanism by which this “synchronous instinct” comes into being and exerts its social function still needs to be investigated.

In the study of animal behavior, this interesting phenomenon of behavioral synchronization is known as a behavioral contagion. Behavioral contagion has been defined as a phenomenon in which the event that an individual engages in an unlearned behavior automatically triggers a similar behavior in others [5], which is generally reserved for motor mimicry of specific self-directed behaviors (SDBs) or social actions [6]. Behavioral contagion involving yawning, scratching, self-grooming, scent-marking and other behaviors have been observed in several primate species (e.g., chimpanzees (*Pan troglodytes*) [7,8,9,10], gelada baboons (*Theropithecus gelada*) [11] and bonobos (*P. paniscus*) [12,13]). Contagious yawning is the primary approach to studying behavioral contagions in humans and other animals.

Higher social relationship quality could promote the occurrence of behavioral contagions. Accordingly, behavioral contagion is more common between intimate partners [4,14]. In the study of yawn contagion, the contagion between human dyads with strong social bonds seems to be stronger [15]. In previous nonhuman primate studies, yawning was more contagious between kin and friends [11,12,13], just as in humans. Unfortunately, some experimental studies failed to provide convincing evidence for yawn contagion in gorillas (*Gorilla gorilla*) [16], stump-tailed macaques (*Macaca arctoides*) [17], ring-tailed lemurs (*Lemur catta*) and ruffed lemurs (*Varecia variegata*) [18]. It has been suggested that yawn contagion is limited to a few species and has evolved independently [19], which illustrates the ongoing debate on the possible mechanisms behind contagious yawning. Thus, it is necessary to study more contagious behaviors and to pursue evidence suggesting their social functions.

Based on the previous hypothesis that yawn contagion between close social partners seems to be stronger and would be related to empathy [20], we speculate that contagious yawning may not be observed among species without strong affiliative tendencies. Some findings in naturalistic and experimental paradigms are consistent with this conjecture; that is, a group of captive western lowland gorillas does not show yawn contagion [21]. This is a result that would be considered reasonable since the sociality of gorillas may be lower compared to other highly social apes (e.g., chimpanzees and bonobos). However, yawn contagion has been found in semi-solitary Bornean orangutans [22], which are the least social great apes. Captive breeding may artificially affect the social connections of the subjects, which produces some unexpected results that may be contrary to the prediction. The relationship between social relationships and behavioral contagion needs to be proved by the discovery in the wild environment.

Behavioral contagion is an effective approach to synchronizing neighboring group members’ activities, which can ultimately promote group cohesion and social coordination. In free-ranging bighorn sheep (*Ovis canadensis*), vigilance behavior is contagious between neighboring rams [1]. Behavioral contagion may influence the stability of the population by making neighbors share the common state. During flight preparation, yawning and stretching can be contagious in a flock of budgerigars (*Melopsittacus undulatus*), and consequently, the group state can be synchronized before collective movement [23]. Recently, scratch contagion has become another attractive part of behavioral contagion study. Scratch contagion has been found during tense situations in Bornean orangutans (*Pongo pygmaeus*) [24]. The captive environment narrows the distance between individuals of this low intimacy species, which may put the whole group under greater pressure. Scratching is usually related to the existence of psychological and physical stresses [25,26]. Scratch contagion may serve to sense the stress level of the social context and reduce possible conflict. Furthermore, social attention may also be one of the keys [27]. Any behavior that increases attention toward an individual has the potential to facilitate behavioral contagion [1,28]. Behavioral contagion may increase social cohesion as a coordinated and adaptive process for group members [24,29,30]. We intend to focus on the spatial distance between the two sides of the contagion and the social context when contagion occurs.

Hitherto, behavioral contagion in macaques has rarely been reported. There are more than 20 species of macaque, which are widely distributed. The research on behavioral contagion, a feature shared by humans and animals. We need to pay attention to this large-scale primate genus. Our study was carried out in a group of free-ranging Tibetan macaques (*M. thibetana*). We focus on scratching because it accounts for the highest proportion of self-directed behavior in this species and has been proved to have a certain social function [24,31]. Yawning should also be noticed, which is the main aspect of behavioral contagion studies. We are committed to exploring whether behavioral contagion is related to social relationships and maintaining social stability. We made predictions and tried to answer them in these respects.

*Predictions 1 and 2*

Tibetan macaques live in multi-male and multi-female groups with moderate size, strong cohesion and sustainable stability and form a matriarchal society [32]. Compared with other primate species with a strong subordination tendency [11,12,13], we predict that (1) Behavioral contagion tended to be stronger between close social partners and kin. (2) Group members with high values of centrality would be involved in more contagious behaviors.

*Prediction 3*

Since behavioral contagion may play a role in synchronizing group members’ status and local stability of the group [1,2,23], we predict that behavioral contagion would be stronger among neighboring group members.

*Prediction 4*

The society of Tibetan macaques is autocratic, in which individuals live in groups under a strict linear ruling hierarchy [33,34]. Scratching and yawning are more common during post conflict reconciliation [31]. Behavioral contagion may exert its effects during this period, thus, avoiding more conflicts. We predict that behavioral contagion would be more frequent among individuals with smaller rank distances because the conflicts between them were easier to reconcile.

## 2. Materials and Methods

### 2.1. Study Site and Subjects

We conducted this study at the “Valley of the Wild Monkeys” (N30°04′25.1″/E118°08′59.3″), Mt. Huangshan National Reserve in Anhui, China. Our study was carried out in a wild population of Tibetan macaques (Yulinkeng, China). The research on the YA1 group has been carried out since 1986, and the changes in all members of the group and the main population dynamics have been recorded [32]. Based on this continuous record, we can identify all individuals in this troop according to their natural characteristics, such as scars, hair color patterns and/or facial/body appearance [35]. In this study, from November 2021 to February 2022, the troop consisted of 48 members, including 12 adult females, 9 adult males, 5 sub-adults, 15 juveniles and 7 infants.

We provided monkeys with 3–4 kg of corn at fixed intervals every day (09:00, 11:00, 14:00 and 16:00) and sprinkled it evenly at a fixed and high visibility place (216 m^2^) to ensure that all monkeys could get it [35,36,37,38]. The study group ranged freely in the forest for most of the day, and the main food source was natural wild plants.

### 2.2. Data Collection and Behavioral Definition

Focal animal sampling [39] of 10 min sessions was used to observe and collect social behaviors data (e.g., grooming, contact sitting and proximity) from 26 adult and sub-adult members (including 9 adult males, 13 adult females, 2 sub-adult males and 2 sub-adult females, see Table S1) in the monkey troop. Observations for the focal group lasted 6.5 h per day from 08:30 to 11:30 and from 14:00 to 17:30. The total focal sampling time of all individuals was 7280 min, with 28 rounds for each individual. Due to the large-scale activities and scattered distribution of the study group in the forest, while conducting focal animal sampling, we used an all-occurrence sampling method [39] to record all scratching and yawning behaviors within a radius of 5 m centered on the focal individual and to record all scratch and yawn contagions within 10 m. For example, when we observed and recorded the focal animal “A”, we may record the 10 m behavioral contagion between “B” in the west 5 m and “C” in the east 5 m and more behavioral contagions in a smaller range. We also used the all-occurrence sampling method to record mating and aggression-submission rounds. All the above behaviors were recorded in the digital voice recorder (AIGO, Beijing, China). Detailed behavioral definitions are shown in Table 1 [24,31,32,40].

Since scratching and yawning of the focal animal subgroup were recorded by the all-occurrence recording method while the focal animal sampling was going on, we could record and identify the following variables when sorting [24]: (a) occurrence time of yawns and scratches; (b) identity of the possible expresser and observer; (c) time latency of adjacent yawns and scratches, in seconds (s); (d) visually estimated distance between expresser and observer (<1 m, 1–3 m, 3–5 m and 5–10 m); (e) the context in which the contagion occurred, categorized as “tense” or “relaxed” (based on aggression and submission that occurs prior to contagion). To ensure the reliability of our data, we limited the dataset to a circular space with a radius of 5 m, without recording the situation where the expresser and observer are blocked by trees or stones or their distance was >10 m.

### 2.3. Data Analysis

#### 2.3.1. Determination of Behavioral Contagion

A total of 328 yawning and 2101 self-scratching behaviors were recorded. We found that the frequency of yawning was lower and mainly repeated by a single individual, and there were only 25 possible contagious yawns available for analysis. Therefore, we had to only focus on the scratch contagion.

Due to the complexity of the sampling environment (e.g., rocks and trees), only the scratch contagion that triggered the behavior and was seen by the observer was recorded, so we could not evaluate the presence of the contagion by comparing it with the baseline [11]. Tibetan macaques have a large range of scratching behaviors, which may attract attention except in the opposite direction. Therefore, during the sampling period, within ten minutes, we pay great attention to the completely backward individuals and do not judge the adjacent scratch of them as contagion. The analyses of contagion followed similar methods to Miller et al. [23] (for budgerigars) and Massen et al. [41] (for marmosets). The time between adjacent scratches was calculated (inter-scratch interval), and the time latency of adjacent scratches was binned into 10 s intervals. Each 10 min observation was broken into sixty 10 s bins. In addition, if a false contagion signal occurs because one individual shows scratches many times in consecutive bins, the observation is deleted from the analysis [41]. Next, according to the method of Campbell, M.W. and Cox, C.R., we judged the adjacent scratches within a specific time span as contagion. Based on the number of scratches in each bin, we calculated the average and standard deviation. In order to establish a threshold of contagion, we used the mean number of adjacent scratches contained per bin as the reference value and added 1.96 times the standard deviation. This yielded a line above which any rate of scratching would have a less than 5% chance of occurring (i.e., *p* < 0.05) on a Z distribution. The judgment of whether the scratch was contagious could be based on whether the rate of scratching exceeded this threshold.

#### 2.3.2. Relationship Quality

We quantified the strength of dyadic social relationship quality with the Dyadic Composite Sociality Index (DSI) [42]. The rate of affiliative behaviors (social grooming, contact sitting and proximity) extracted from the focal-animal observations was used to calculate the DSI scores. The calculation formula is as follows:

$$DSI_{xy}=\frac{\sum_{i=1\frac{f_{ixy}}{{\overset{=}{f}}_{i}}}^{d}\;}{d}$$

where *d* is the number of behaviors that contribute to the index; $f_{ixy}$ is the rate of behavior *i* for dyad *xy*; and $\overset{=}{f_{i}}$ is the mean rate of behavior *i* across all dyads in our study group [43].

Different from the study in which objects are same-gender [42], there was a considerable number of males (*n* = 11) and females (*n* = 15) in our study group, and there were obvious gender deviations in the rate of homosexual mounting, hold bottom, embrace and other behaviors in this species [32], so these behaviors were not taken into account in the formula. We calculated the DSI scores of 256 dyads. The values of this index range from 0 to ∞. High values of the DSI represent dyads that have more frequent and/or longer-lasting affiliative interactions than the average dyad in their group, and low values of the DSI represent dyads in the opposite [43].

Furthermore, we identified dyads belonging to the same matrilineal group as relatives [44]. Based on the aggression-submission bouts and win-lose proportions, we calculated David’s Score (DS), which was then used to arrange individuals’ dominance ranks [45]. Individuals with higher DS values occupied higher ranks.

### 2.4. Statistical Analysis

The generalized linear mixed model (GLMM) was established to predict the effect of various factors on behavioral contagion. We used the identity of the expresser and observer as the unit of analysis and collected the times of scratch contagion of each dyad. Then we added these units to the model as random factors. Then, we calculated relationship quality (DSI scores), sex-combination (categorical; take the same gender as “0” and the different gender as “1”), kinship (categorical; take nonrelatives as “0” and relatives as “1”) and rank distance (from 1 to 25) of each dyad and used them as fixed factors. R v4.04 was applied to carry out the analysis for all the above data. The GLMM was run with the counting vector “times” as the dependent variable by using the lme4 package. 

Based on the DSI score matrix, we calculated the eigenvector centrality coefficient of each individual and then drew the social network by NetDraw v2.084. GraphPad v8.02 was used for drawing other figures.

## 3. Results

### 3.1. Scratch Contagion Analysis

**Figure 1:**
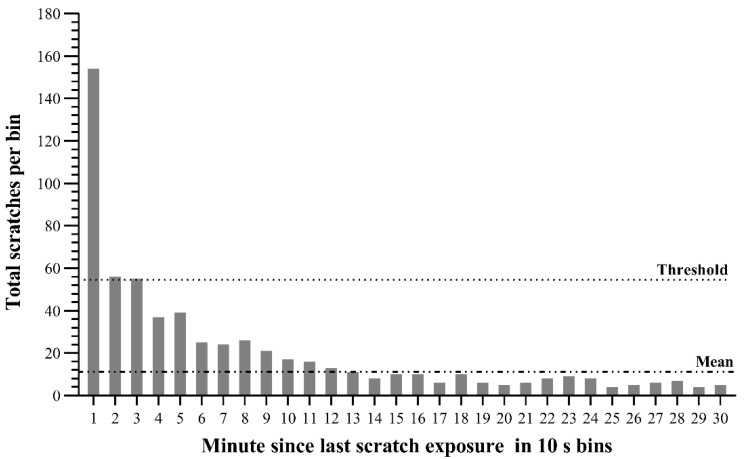
A graph of the number of scratches per 10 s bin. The mean and standard deviation were calculated from all bins (up to minute 10), only showing the first 5 min for clarity. Before 300 s passed, the strength of scratch contagion decreased significantly with time delay, and this decline continued after that. Contagion occurs quickly after seeing the triggered scratch and lasts until 30 s after the triggered scratch.

With a mean of 11.033 scratches per bin and a standard deviation of 22.217, the threshold for evidence of contagion was 54.578 yawns in a bin. Examining Figure 1 with the criteria described above, we found evidence for contagion starting immediately after seeing a scratch and lasting for 30 s. The following data analysis was based on 265 scratch contagions occurring within 30 s.

### 3.2. Factors Affecting Frequency of Scratch Contagion

**Figure 2:**
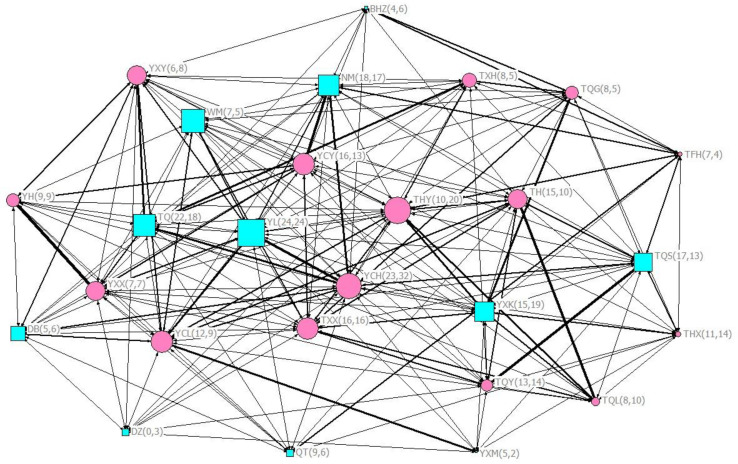
Eigenvector centrality coefficients of group members in the social network. Nodes represent all individuals in the group; males and females are respectively shown in blue squares and orange circles; node size is directly proportional to centralities; line thickness represents members’ association index in the social network; the letters on labels represent the name of each individual, and the number after each individual’s name represents the times it has become expresser and observer, respectively.

A social network was drawn on the data for two months, intuitively showing the interaction between group members and the occurrence of scratch contagion during this period (Figure 2).

The GLMM displayed that relationship quality (*p* < 0.001, coeff ± SE = 0.019 ± 0.002) had significant effects on scratch contagion (Table 2). Sex-combination, kinship and rank distance did not affect scratch contagion.

### 3.3. Strength of Scratch Contagion Changed with Distance between Expresser and Observer

**Figure 3:**
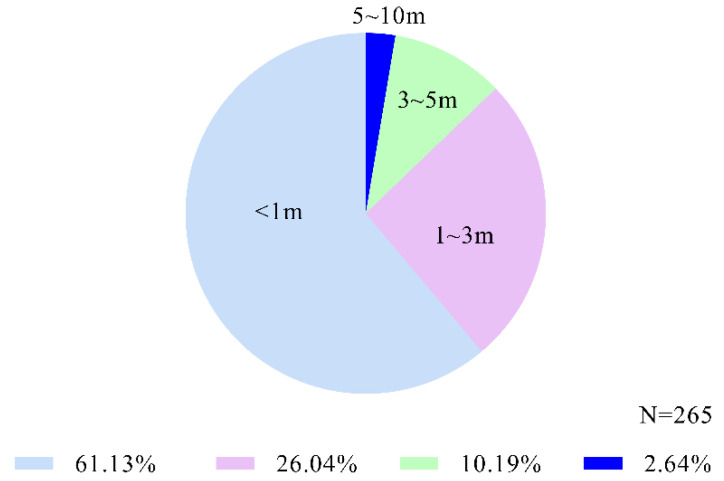
Distribution of scratch contagion at different distances.

In our scratch contagion records (N = 265), 162 times occurred within 1 m, accounting for 61.132%; 69 times occurred within 1–3 m; 29 times occurred within 3–5 m; and 7 times occurred within 5–10 m (Figure 3). The strength of scratching contagion was higher in individuals with smaller spatial distances.

## 4. Discussion

The recent study aims to investigate whether behavioral contagion has certain social functions and its link to empathy. Behavioral contagion seems to be a unique phenomenon that contributes to empathy. In the framework of the “Russian Doll” model [20], the perception-action mechanism, which is the cornerstone of empathy, explains the most basic emotional reactions, such as mimicry and behavioral contagion [6]. Based on research findings, we summarized and next showed relevant evidence to support the link between behavioral contagion and empathy and suggested the social function of behavioral contagion.

We provided evidence of the spontaneity of a behavioral contagion, which can support the presence of scratch contagion in Tibetan macaques. The perception-action coupling mechanism dominated by the mirror neuron system is the physiological basis of behavior contagion [46,47]; that is, animals may produce similar behaviors in a short time when they see some familiar behaviors of others. We found that scratch contagion tended to occur in a very short time, and it was less likely to occur with time delay (see Figure 1). It should be noted that our judgment of scratch contagion is based on each pair of adjacent scratches, but we have to admit that some contagions may be affected by triggered scratches longer ago. This is an unavoidable limitation of behavior observation experiments. Compared with previous studies, this highlights the instantaneity of scratch contagion in Tibetan macaques. In the yawn contagion study of gelada baboons, even if it has been proved that there is a significant difference between the yawn contagion within five minutes and the baseline situation of a spontaneous yawn, the contagion occurs more in the second minute, and its frequency in the other four minutes is at an approximate level [11]. Such instantaneity was also not shown in the study of scratch contagion in orangutans [24]. In the pair-housed cage experiment, there are scratch contagions concentrated in the first 60 s [48], but they may be subjected to unavoidable human interference, such as the increased stress due to a small-scale cage. Just like rapid facial mimicry (RFM) [49], contagious scratching may be an automatic, unconscious and rapid motor mirror reaction, which is conducive to the emotional communication between members of the group. By feeling the emotional activities of others, costly interaction and even conflict can be avoided [50]. However, the link between behavioral contagion and empathy also needs to be reflected in the strength change of contagions between different individuals because the relationships between members are not at the same level.

Behavioral contagion may build a bridge of empathy. So far, the potential link between behavioral contagion and empathy has not been directly proved, but some types of behavioral contagion are thought to reflect empathy because they are related to emotional affinity and perspective-taking [6]. In our study, scratch contagion was stronger among members with high-quality social relationships, which provides more support for the relationship between behavioral contagion and empathy. As positive feedback, behavioral contagion may contribute to strengthening the social relationship between group members. The individuals whose own behavior is repeated will recognize this change and be more intimate with imitators [51,52,53]. Prediction 1 that behavioral contagion tended to be stronger between close social partners was supported. However, contagions were not stronger among related individuals. This may be due to the small proportion of kin dyads in all dyads (43 in 325). Therefore, we cannot deny the influence of kinship on behavioral contagion, which needs more rigorous experiments to prove. We recorded scratch contagions by observing focus subgroups in the range of 10 m, but it is difficult to pay attention to those outgroup members who tend to deviate from the center of the group. This is unfair to these few individuals, who are alienated from others and have fewer recorded scratch contagions. It is difficult to control the time of each individual in the focus subgroup at a similar level, and it is impossible to record contagion on a larger scale because they may not be able to sense others’ presence. Therefore, we tried to reduce the bias in recording contagion by avoiding focusing on subgroups with fixed members. Based on the social grooming and proximity frequency of all individuals, we quantified the relationship quality by calculating the DSI score of each dyad. This is different from the direct analysis of the impact of grooming frequency on behavioral contagion [11]. The relationship quality score was calculated by comparing it with the average social interaction level of the whole group. Therefore, although more rigorous relevant experiments are needed to prove this correlation, our observation process and results are relatively scientific.

We also found that scratch contagion occurred more frequently between adjacent individuals. The spatial proximity effect of behavioral contagion may be a part of the feedback loop to maintain group cohesion [1]. Most scratch contagions occurred when individuals were within 1 m (>60%). Prediction 3 that behavioral contagion would be stronger among neighboring group members was supported. This may involve social concerns that are necessary to coordinate behaviors and maintain close relationships with group members [54]. However, it should be noted that there is confusion between spatial proximity and emotional proximity. We observed that more and faster scratch contagions occurred when individuals sat next to each other and after social grooming paused, and usually after contagions occurred, they would start or continue social grooming and other friendly behaviors. Based on our observation method, we may record more contagions between adjacent individuals in this case. Scratching and yawning are more common during post conflict reconciliation [31]. For these reasons, we rarely observed the contagion in the tense social environment and did not analyze this variable. Behavioral contagion may be associated with the ability of animals to maintain friendly social interaction and avoid conflicts. It may reflect their visual attention to surrounding friends [27] or understanding of others’ emotions [20]. Thus, it will be more likely to detect their scratches in the first place. From a group perspective, this frequent behavioral contagion between neighbors is an important aspect of group life because it helps to synchronize the activities of group members [1]. This may enable them to be stabilized in the common groupuscule.

This group maintenance function is also reflected in those group center members. We showed the distribution of behavioral contagions among group members through the social network. Intuitively, members with higher values of centralities form the core of social groups, and they tend to become expressers and observers of contagion more frequently. Prediction 2 that group members with high values of centrality would be involved in more contagious behaviors was supported. In addition, the female named YCH and the male named YL had the highest centrality. It is worth noting that the male led the whole monkey group (rank = 1), while the female was not in the top quarter rank (rank = 8), but she formed a stable consortship with the male, and both of them have participated in the most behavioral contagions. In Tibetan macaques, individuals with high social centrality play a central role in the group. For example, they send more visual signals in group movement so as to promote collective decision making [55]. Core members may pay attention to outgroup members and supervise the situation of the group. This may also be evidence that behavioral transmission is related to social attention. However, individuals with higher values of centrality may be looked at more frequently, and thus contagion will be more likely simply as a result of differences in scratch detection [56]. In the field observation, although we have achieved the random sampling of focus animals, we cannot interfere with the existence of surrounding individuals. Therefore, this result needs more variable control experiments to prove.

## 5. Conclusions

We obtained some novel findings about the presence of scratch contagion among wild macaques and the possible link between scratch contagion and empathy. Scratch contagion is stronger in monkey dyads with higher social relationship qualities. Contagions occur frequently and centrally among members at the center of the social group. Behavioral contagion may strengthen the combination between group members through an empathy connection and strengthen group cohesion to maintain the stability of the whole social group. The presence of scratch contagion in Tibetan macaques and its relevancy with the strength of bonding between individuals suggest that this species may present the basic components of the multilayered empathy which has been shown in humans and anthropoids. For the study of empathy evolution, primate behavioral contagion research should be carried out on a larger species span.

## Figures and Tables

**Table 1:** Ethogram of behaviors recorded during focal animal samples.

Catalog	Definition
Behavioral contagion	Self-scratching and yawning of individuals whose own behavior is repeated by other individuals for some time; the former is called the expresser, and the latter is called the observer.
Self-scratching	Movement of the hand or foot during which the fingertips are drawn across fur or skin. Not recorded as self-scratching if accompanied by fiddling with the fur carefully.
Yawning	Brief gaping movement of the mouth. Not recorded as yawning if accompanied by aggressive signals such as eye flash or canine whetting.
Some other social behaviors	
Social grooming	One individual orally or manually manipulates the fur of another.
Contact sitting	Two or more individuals are sitting or huddling in close body contact lasting more than 5 s.
Proximity (<1/3/5 m)	Two or more individuals keep a sitting or lying posture within a certain distance. In this study, the distances of 1, 3 and 5 m were recorded, respectively.
Aggression	An individual stares, hits on the ground, chases or orbits another individual.
Submission	An individual is attacked by another, but quickly leaves or flees in the opposite direction.

**Table 2:** Results of GLMM (family = Poisson) used to test the factors affecting the scratch contagion.

Factors	Coefficients	SE	Z	*p*
Relationship quality	0.019	0.002	8.49	<0.001
Kinship	−0.328	0.203	−1.62	0.105
Sex-combination	0.128	0.142	0.901	0.368
Rank distance	0.125	0.301	0.415	0.367

## Data Availability

Not applicable.

## Supplementary Materials

The following supporting information can be downloaded at: https://www.mdpi.com/article/10.3390/ani12162151/s1, Table S1. Characteristics of focal Tibetan macaques in the Yulinkeng 1 (YA1) group during observation.
