# Supplementary material for: “Emotional Proximity” and “Spatial Proximity”: Higher Relationship Quality and Nearer Distance Both Strengthen Scratch Contagion in Tibetan Macaques
Source: Animals (Basel). 2022 Aug 22;12(16):2151. doi: 10.3390/ani12162151 (PMC9404782; doi:10.3390/ani12162151)
Supplement: Supplementary file 1 [file animals-12-02151-s001.zip › Table S1.pdf]

ID (adults and sub adults, a,b and c mean three matrilineal)	Age	Sex	Number of relatives	Social rank
YL	8	Male	0	1
YXK[a]	9	Male	6	2
YXX[a]	11	Female	6	3
YH[a]	18	Female	6	4
DB	8	Male	0	5
NM	9	Male	0	6
TQ	11	Male	0	7
WM	15	Male	0	8
YCY[a]	13	Female	6	9
YXY[a]	6	Female	6	10
YCH	9	Female	0	11
TXH[b]	12	Female	6	12
YCL[a]	9	Female	6	13
DZ	7	Male	0	14
TQS[b]	6	Male	6	15
BHZ	12	Male	0	16
TH[b]	18	Female	6	17
TXX[b]	13	Female	6	18
TQL[b]	8	Female	6	19
THY[c]	12	Female	2	20
TQY[b]	5	Female	6	21
QT	7	Male	0	22
THX[c]	9	Female	2	23
YXM[a]	5	Male	6	24
TFH[c]	5	Female	2	25
TQG[b]	4	Female	6	26
